# The paradigm shift in allergy consultations through a digital ecosystem

**DOI:** 10.3389/fdgth.2024.1402810

**Published:** 2024-04-25

**Authors:** Inmaculada Sánchez-Machín, Paloma Poza-Guedes, Elena Mederos-Luis, Ruperto González-Pérez

**Affiliations:** ^1^ Allergy Department, Canary Islands University Hospital, Santa Cruz de Tenerife, Spain; ^2^ Severe Asthma Unit, Canary Islands University Hospital, Santa Cruz de Tenerife, Spain

**Keywords:** digital health (eHealth), digital ecosystem, e-consultations, management of waiting lists, allergy, information and communication technologies (ICTs)

## Abstract

In Spain, specialist outpatient care traditionally relied on in-person consultations at public hospitals, leading to long wait times and limited clinical analysis in appointment assignments. However, the emergence of Information and Communication Technologies (ICTs) has transformed patient care, creating a seamless healthcare ecosystem. At the Allergy Department, we aimed to share our experience in transitioning form a traditional linear model of patient flow across different healthcare levels to the implementation of a digital ecosystem. By telemedicine, we can prioritize individuals based on clinical relevance, promptly and efficiently addressing potentially life-threatening conditions such as severe uncontrolled asthma or hymenoptera venom anaphylaxis. Furthermore, our adoption of telephone consultations has markedly reduced the need for in-person hospital visits, while issues with unstable patients are swiftly addressed via WhatsApp. This innovative approach not only enhances efficiency but also facilitates the dissemination of personalized medical information through various channels, contributing to public awareness and education, particularly regarding allergies. Concerns related to confidentiality, data privacy, and the necessity for informed consent must thoroughly be addressed. Also, to ensure the success of ICT integration, it is imperative to focus on the quality of educational information, its efficient dissemination, and anticipate potential unforeseen consequences. Sharing experiences across diverse health frameworks and medical specialties becomes crucial in refining these processes, drawing insights from the collective experiences of others. This collaborative effort aims to contribute to the ongoing development of a more effective and sustainable healthcare system.

## Introduction

Allergic disorders persist as major contributors to global morbidity, with a noticeable rise in both total prevalence and incidence worldwide (1). Meeting the increasing demand for allergy-related healthcare services highlights the importance of proper efficiently allocating both professional and physical resources.

Information and Communication Technologies (ICTs) offer clear benefits in enhancing connectivity among different levels of healthcare professionals and patients, as well as in managing waiting lists for medical services. These aspects are integral to political and healthcare debates in most first-world countries, serving as a metric for political management. Like many disruptive changes, the advantages of ICTs are so apparent that their adoption has rapidly occurred without a predefined roadmap.

**Figure 1:**
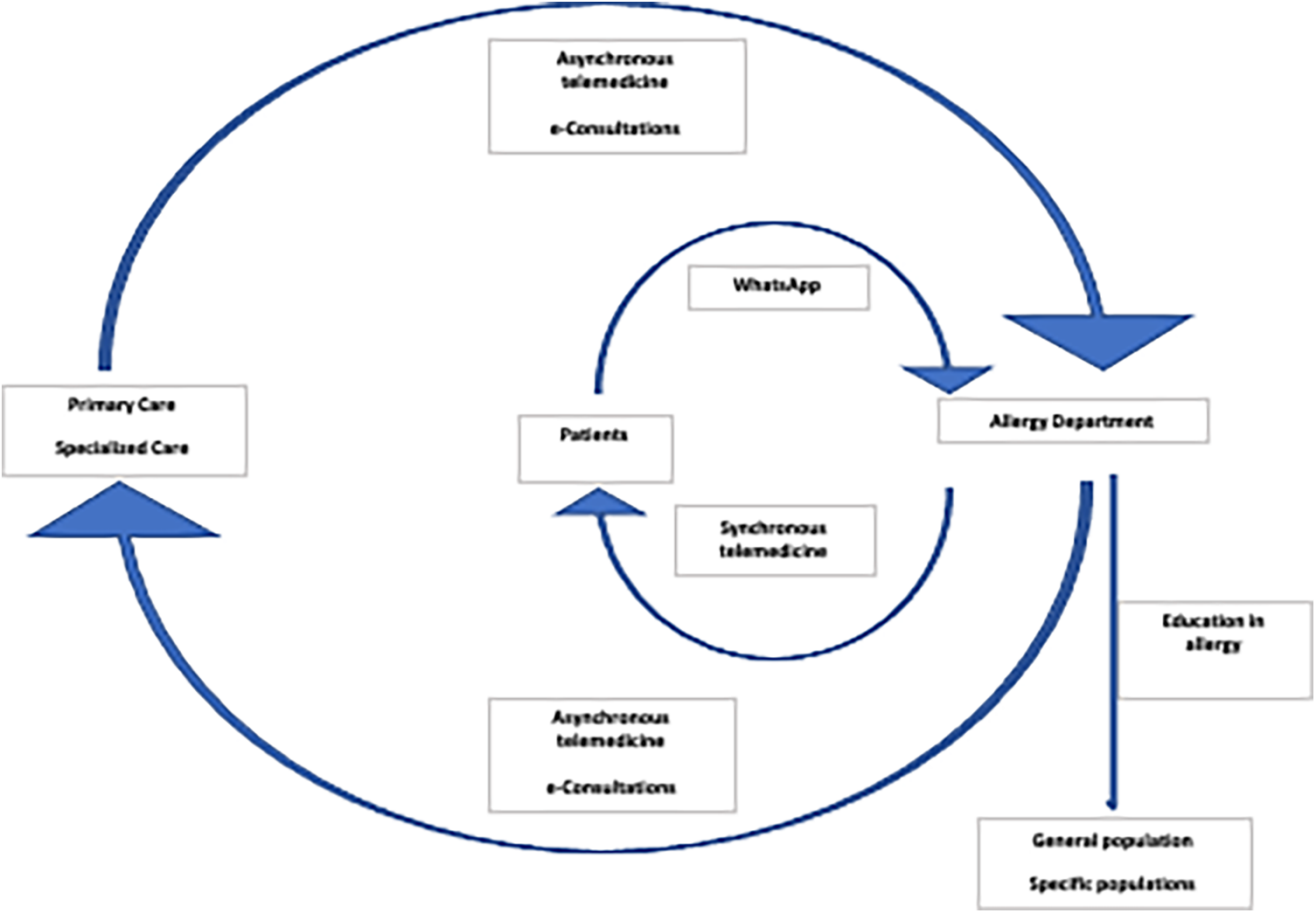
Digital ecosystem: Medical information flows nonlinearly through essential information and communication technologies like asynchronous telemedicine with physicians, synchronous telemedicine with patients, social media, WhatsApp, and educational channels encompassing both healthcare providers and users.

The connectivity provided by ICTs, has transformed the traditional linear structure of patient flow from primary to a more complex healthcare level. Traditionally, medical consultations at the Allergy Department of the Hospital Universitario de Canarias, a public tertiary level care institution in Tenerife, Spain, were conventionally managed through a paper-based system. Medical appointments were scheduled on a first-come, first-served basis, neglecting individualized clinical aspects or potentially associated risk factors. Since 2013, this approach has evolved into a circular paradigm (Figure 1), and we now operate within an interconnected system, shaping a genuine ecosystem where information flows nonlinearly through the following essential ICTs:

• Asynchronous telemedicine with physicians.
• Synchronous telemedicine with patients.
• Social media.
• WhatsApp.
• Educational channels.

In this paper, we delineate the outcomes after the incorporation of a digital ecosystem, examining the network of interrelationships and the utilization of ICTs at our institution, catering to a population of 498,208 healthcare system users.

## Asynchronous telemedicine with physicians (e-consultations)

Since 2017, our institution's Allergy Department has transitioned all consult request from paper-based to electronic consultations (eC), defined as bidirectional, asynchronous, text-based provider-to-provider queries (2). In 2022, out of a total of 6,331 e-consultations, 5,273 (83%) were originated from Primary Care (PC), with the remaining 1,058 (17%) were referred from Specialty Care (SC). Participant Allergists are required to use a personalized username and password, digitally sign each comment, and committed to respond each eC within 24–72 h. All eC, regardless of their outcome -even those that do not result in a physical consultation to the Allergist- are securely documented in the patient's EMR, guaranteeing accessibility for both PC and SC providers to review and analyze the e-consultations details.

**Figure 2:**
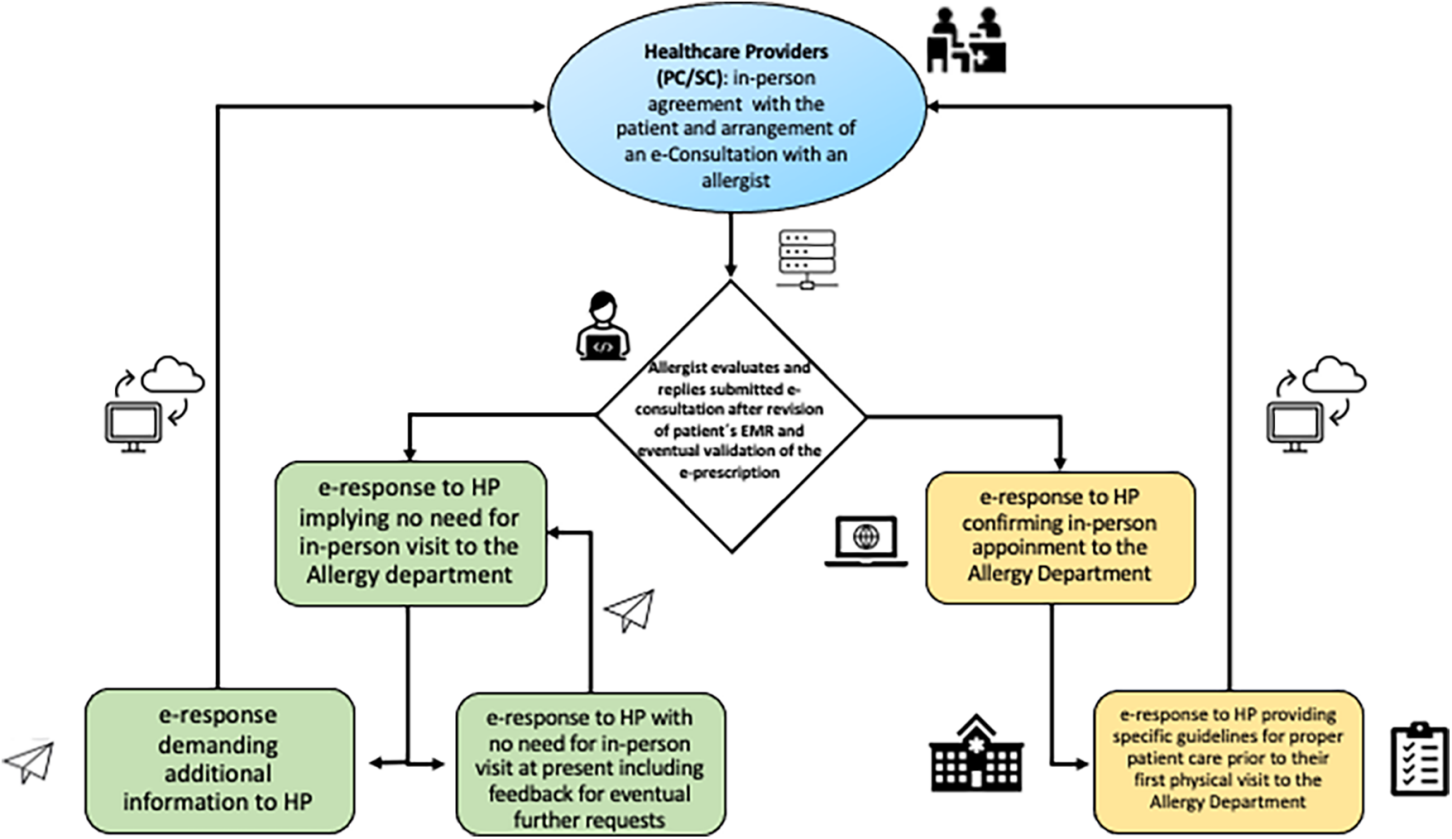
Documented pathway of a digital ecosystem at the Allergy Department. EMR, electronic medical record; HP, healthcare providers; PC, primary care; SC, specialist care; e-Consultation, electronic consultation.

This process enables us to prioritize the Allergy Department waiting list focused on the severity of the referred clinical condition. It ensures that patients with life-threatening conditions -i.e., hymenoptera venom anaphylaxis, severe uncontrolled asthma, or food and/or drug anaphylaxis- can receive a scheduled in-person consultation within 24–72 h. Moreover, to enhance the efficiency of face-to-face consultations, specific instructions like discontinuing oral antihistamines 7–21 days before a skin prick test with aeroallergens can be communicated in advance through an intended eC (Figure 2).

In 2013, 12.12% of Allergy Department eC were remotely -i.e., that the eC were successfully addressed without the need for an in-person visit to the Allergy Department- resolved (3). Currently, as of 2022, 42.5% of eC from PC and 39% eC from SC (2,623 patients), no longer require a face-to-face appointment, in 2023, 42.8% of eC from PC and 36% eC from SC (2,529 patients), did not require a face-to-face appointment, showcasing the growing impact of telemedicine in minimizing unnecessary in-person visits and optimizing the allocation of healthcare resources. This trend highlights telemedicine as a transformative force, shaping the future of healthcare accessibility and patient-centered care.

This process has inherent limitations, including patient preferences. Some individuals may prefer direct, face-to-face consultations with specialist rather than relying on their family physicians as intermediaries. Additionally, the transmission of information through mediators, even if they are physicians poses a challenge. Written information may not consistently convey the nuanced details that specialist require for a comprehensive understanding of a patient's condition.

## Synchronous telemedicine with patients

Telemedicine sessions with patients involve direct synchronous interaction between the allergist and the patient, presently facilitated through telephone calls. Following the national COVID-19 lockdown emergency in Spain, telephone medical consultations increased from 0% to 91.14% in just 4 days at the Allergy Department (4). The rapid shift to telemedicine, driven by COVID-19 precautions, lacked prior preparation but resulted in a highly rated overall patient satisfaction (5).

Despite post-COVID pandemic, face-to-face consultations have resumed, telephone consultations remain still active for specific situations (Supplementary Table S1):

• Out of a total of 7,704 annual consecutive consultations in 2022, 644.06 (8.7%) were conducted via telephone, while similarly in 2023, this method accounted for 8% (569.76) out of 7,122 consecutive consultations received by the Allergy Department. All telephone consultations (100%) were related to deal with elements such as laboratory and/or imaging results and/or tracking patients’ progress in their individualized scheduled allergen immunotherapy (AIT) regimes. It should be noted that patients requiring *in vivo* procedures such as spirometry and/or skin testing are scheduled for and undergo these tests on the same day, in advance of their follow-up telephone consultation.
• Annually, the nursing staff oversees 880 appointments for drug and/or food oral tests, ensuring patients are in optimal conditions upon arrival.
• Precise monitoring of delayed reactions following food/drug allergy challenges or the administration of biologics or AIT.

The information collected in the patient's electronic medical record (EMR), upholds the same standards of quality, ethics, and security as face-to-face consultations. Teleconsultation guidelines are present in certain medical specialties, though not universally across all (6). In our experience, the following specific skills are essential:

• Introduce yourself briefly.
• Verify the patient's identity to respect confidentiality.
• Confirm subject's preparedness for the call, acknowledging that pre-scheduled appointments may unexpectedly “catch” the patient by surprise.
• Conduct the interview efficiently to gather key information.

Although technology allows for remote physical exams and mobile health (mHealth) provides allergists with daily clinical data, our healthcare area has yet to incorporate these practices (7). Successful implementation not only necessitates possessing the required devices but also adapting them to integrate seamlessly with the in-house EMR system. Also, a protocol should be established for documenting work when the patient is unreachable, including the number of phone call attempts to be made.

## Social media: health information

While information from internet-based sources is gaining importance, there are currently no standardized methods to assess the quality of medical information on social networks (8). The use of social media by allergists at conferences in various countries, including the USA and Spain has significantly risen (9). Nevertheless, informational endeavors in this domain are currently voluntary and not formally recognized or acknowledged at our institution.

Since 2013, we operated a webpage with static information and email contact (10). From March 2017 to November 2020, approximately 2,400 patients have been informed about the existence of our website. Currently, the *Facebook*-group “*alergia-vacunas*” have 441 members. Our website www.alergia-vacunas.es, receives an average of 2,205 visits per month. However, we receive only 29 emails from 6 patients, 6 WhatsApp chats, and 3 Facebook messages monthly, none of which involve inappropriate inquiries or offensive messages. Regrettably, in late 2020 the webpage was discontinued due to financial difficulties in consistently updating its scientific content, and presently our scientific knowledge dissemination takes place through the hospital press section, encompassing 6 press publications, 6 radio interviews, and 11 television appearances since 2022. Although a direct comparison in terms of efficacy has not been feasible after the discontinuation of the website, the expected ease of access provided by a website is currently lacking. This situation is likely to specially affect young adults (18–34 years old) or those individuals seeking professional medical information online (11).

## Whatsapp

WhatsApp serves two primary functions within our practice:

• **Group Communication:** Engaging all Allergy Department staff to facilitate organizational task and information sharing.
• **Patient Communication:** Particularly to individuals managing different clinical conditions. This encompasses, on average, 19 patients per month with severe uncontrolled asthma despite biologic therapy, 12 individuals undergoing active food desensitization protocols at home, and 18 subjects with unstable rare diseases -like hereditary angioedema- throughout 2023.

Participation in WhatsApp is optional for both allergists -three out of 6 (50%) Allergist at our institution agreed to provide WhatsApp follow-up- and patients, and currently takes place without obtaining written informed consent. While some authors argue that sharing a cellphone number implies tacit consent, the patient must explicitly agree to dispense information through a social networking app with the physician, acknowledging that data may be stored on the physician's cellphone for a specific period (12, 13).

The use of WhatsApp follow-up was limited to specific patients diagnosed with hereditary angioedema, those with unstable severe asthma and individuals undergoing active food desensitization protocols at home. This aids in addressing concerns about related adverse reactions and allows for dose adjustments at home (14). However, this imposes an additional responsibility on allergists since WhatsApp functions occur “on demand” without prior scheduling. Interestingly, despite the patient's abuse of this form of direct communication with the physician may be considered a potential drawback beforehand, no such occurrences have been registered so far and, in our experience, WhatsApp texting for acute concerns has enabled us to promptly guide patients to acute treatment (i.e., the rapid use of self-administered adrenaline) and/or specifically seeking emergency care. Moreover, despite not quantified the use of follow-up visits through WhatsApp has expanded the intervals of in-person visits in selected clinically stable patients, reducing their number of work or schooldays missed.

## Education

Despite the prevalence of allergies, there is limited investment in continuous medical allergy education. Since 2013, we have conducted an annual face-to-face allergology educational activity for primary care physicians, attracting an average of 60 participants each year (15). Additionally, since 2016, our Allergy Department has also coordinated an ongoing online certified educational program for schoolteachers covering the region (Canary Islands), focusing on the management of food allergies and asthma. From May 2016 to June 2020, over 1,748 educators participated, achieving a 98.5% satisfaction rate among those who completed the intended training (16).

## Discussion

Delving into a local experience within a middle-income country as Spain, we assert that our findings hold broader relevance across various settings. Despite the World Allergy Organization's recommendation of having 1 allergist per 50,000 inhabitants, indicating a requirement of 10 allergist based on our local population, the availability in our institution stands at only 6 specialists at present (17).

In our view, the current experience, originating from a real-world context emphasize the effectiveness of ICTs support beyond controlled experimental conditions. Within a national public healthcare system prioritizing free and universal access, hinging on highly qualified specialists, the susceptibility to waiting lists accentuates the importance of effective management. Regarding each eC as an educational opportunity, interprofessional telemedicine enables us to prioritize and reduce waiting lists, thereby improving the efficiency of first-time consultations. In fact, the waiting time data from 2023 revealed that, although direct evidence on waitlist reduction is lacking, a comparison with other geographically related tertiary hospitals without the use of telemedicine, indicated promising outcomes (18). Moreover, while the NHS in the UK aims to decrease in-person visits over the next five years, previous studies indicate that 34%–92% of specialist referrals did not require face-to-face appointments, and 27% of these referrals may not occur without this form of consultation (19, 20).

In our practice, a rapid response to e-consults is crucial, ensuring that patients are assessed within 24–72 h and no life-threatening cases are delayed. Interestingly, the allergist's expertise is a key factor; those with less experience or a heavier daily workload tend to schedule more in-person appointments. A noticeable gap in current literature is the absence of comprehensive studies that systematically compare outcomes, including time differences, between telehealth and traditional in-person healthcare (5, 20). While telemedicine may not necessarily save specialist time, it does mitigate the necessity for patients to travel. Furthermore, the comparability of patient-physician satisfaction and disease control in allergic conditions to in-person visits also requires further validation (21).

It could be also speculated that easier access to specialist advice may lead to increased referral rates to SC by PC providers. Despite this may improve patient care by facilitating timely diagnosis and management, it could also increase specialist workload, potentially leading to longer wait times and compromising patient care if specialist resources are overwhelmed. In our experience, e-consultation has enhanced access to allergy care and reduced unnecessary physical hospital visits. However, balancing access with effective resource allocation is essential to ensure the standards of high-quality patient care (22, 23). In addition, it may be of interest to mention that currently, and unlike in private practice, the Spanish public healthcare system does not designate specific earnings for physicians conducting telehealth consults with patients in our jurisdiction.

Providing individual patient education in face-to-face sessions is costly, and although some patients prefer personalized learning, reaching a larger audience is essential (24). To address this, we currently offer on-line courses for teachers, doctors, and utilize media platforms, collaborating with medical and patient associations for effective educational strategies (16, 17).

WhatsApp, a widely adopted and disruptive ICT in healthcare, operates without established guidelines (25). The simplicity, immediacy, and cost-effectiveness of WhatsApp have prompted its adoption, highlighting a prevalent lack of consideration for legal implications. In line with former research and based on our experience, ensuring the security of patient data requires transferring all information to the EMR and promptly removing it from WhatsApp (26). Raising awareness about this issue is crucial, emphasizing that the adoption of technology should align with the needs of patients (27).

Leveraging fundamental ICTs, we have implemented a system facilitating nearly immediate interaction between patients and physicians, resulting in elevated care quality, shortened waiting times, increased specialty appointments for priority cases, and decreased unnecessary travel. Essentially, it establishes a more efficient healthcare system. Despite these advancements there are still noteworthy gaps in the application of ICT to medicine that require focused attention. In this regard, several limitations should also be addressed. Firstly, although previous studies have identified the value of telemedicine in clinical practice, the present investigation lacks specific quality assurance metrics to demonstrate the comparability of care provided to patients identified as non-requiring in-person visits to what would have been offered during a physical consultation (28, 29). Secondly, the preferences or concerns of both PC and SC physicians regarding the implementation of this digital process have not been considered, thus hindering our capacity to gain insights into their perspectives regarding on this mode of patient care delivery.

In the context of Health 4.0, fostering extensive collaboration between consumers and providers is essential, with active involvement from patients (28, 30). The significance of sharing experiences across diverse medical settings and specialties remains vital for building a robust groundwork for future applications in varying healthcare systems.

## Conclusions

We are presented with the opportunity to optimize the use of limited healthcare resources and enhance access to specialized care through ICT, mitigating patient barriers related to distance and time. This proactive approach can position allergology for potential future specialist shortages, contribute to remote care, minimize unnecessary travel, reduce the carbon footprint, and elevate the overall allergy knowledge. Progress in this direction rely on the pivotal role of digital health solutions, recognizing the intricate relationship between digital health and healthcare quality. This comprehensive approach ensures that the incorporation of digital health solutions is not merely a technological advancement but a strategic pathway to achieving positive and measurable enhancements in the overall healthcare experience for patients.

## Data Availability

The data that support the findings of this study are available from Servicio Canario de Salud, however, restrictions apply to the availability of these data, which were used under license for the current study, and so are not publicly available. Data are however available from the authors upon reasonable request and with the permission of Servicio Canario de Salud.
