# Supplementary material for: The paradigm shift in allergy consultations through a digital ecosystem
Source: Front Digit Health. 2024 Apr 25;6:1402810. doi: 10.3389/fdgth.2024.1402810 (PMC11079116; doi:10.3389/fdgth.2024.1402810)
Supplement: Supplementary file 1 [file Table1.docx]

**Supplementary Table:** Telemedicine in the Allergy Department: Overview of current telephone consultation types.

| **Electronic Medical Record** | **Telephone Consultation Type** | 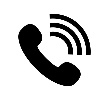 | **Actions** |
| --- | --- | --- | --- |
|  | Subsequent medical consultations | Allergist🠚Patient | Provide the results of complementary tests (laboratory, imaging tests), assessment of clinical progress and/or if necessary, renew prescriptions |
|  | Appointments to carry out individualized food/medication studies | Nursing staff🠚Patient | Confirm patients’ attendance in advance under proper baseline conditions concerning present intercurrent illnesses and/or medication that may interfere with the planned study |
|  | Monitoring of potential late-phase adverse reactions after food/drug challenge tests, or administration of allergen immunotherapy or biologics | Patient🠚Nursing staff or Allergist | Ensure the provision of personalized information concerning potential late-phase reactions |
